# Sigma70Pred: A highly accurate method for predicting sigma70 promoter in *Escherichia coli* K-12 strains

**DOI:** 10.3389/fmicb.2022.1042127

**Published:** 2022-11-14

**Authors:** Sumeet Patiyal, Nitindeep Singh, Mohd Zartab Ali, Dhawal Singh Pundir, Gajendra P. S. Raghava

**Affiliations:** ^1^Department of Computational Biology, Indraprastha Institute of Information Technology Delhi, New Delhi, India; ^2^Department of Computer Science and Engineering, Indraprastha Institute of Information Technology Delhi, New Delhi, India

**Keywords:** sigma70 factor, promoter, machine learning, transcription, prokaryotic genome

## Abstract

Sigma70 factor plays a crucial role in prokaryotes and regulates the transcription of most of the housekeeping genes. One of the major challenges is to predict the sigma70 promoter or sigma70 factor binding site with high precision. In this study, we trained and evaluate our models on a dataset consists of 741 sigma70 promoters and 1,400 non-promoters. We have generated a wide range of features around 8,000, which includes Dinucleotide Auto-Correlation, Dinucleotide Cross-Correlation, Dinucleotide Auto Cross-Correlation, Moran Auto-Correlation, Normalized Moreau-Broto Auto-Correlation, Parallel Correlation Pseudo Tri-Nucleotide Composition, etc. Our SVM based model achieved maximum accuracy 97.38% with AUROC 0.99 on training dataset, using 200 most relevant features. In order to check the robustness of the model, we have tested our model on the independent dataset made by using RegulonDB10.8, which included 1,134 sigma70 and 638 non-promoters, and able to achieve accuracy of 90.41% with AUROC of 0.95. Our model successfully predicted constitutive promoters with accuracy of 81.46% on an independent dataset. We have developed a method, Sigma70Pred, which is available as webserver and standalone packages at https://webs.iiitd.edu.in/raghava/sigma70pred/. The services are freely accessible.

## Introduction

Promoters and enhancers regulate the fate of a cell by regulating the expression of the genes. Promoters are generally located at the upstream of genes’ transcription start sites (TSS) responsible for the switching on or off the respective gene. In prokaryotes, promoters are recognized by the holoenzyme, which is made up of RNA polymerase and a related sigma factor. There are various types of sigma factors responsible for different functions, such as sigma54 controls the transcription of genes responsible for the modulation of cellular nitrogen levels, sigma38 regulates the stationary phase genes, sigma32 regulates heat-shock genes, and sigma24 and sigma18 controls the extra-cytoplasmic functions (Paget, 2015). The number associated with each sigma factor represents the molecular weight. Sigma70 factor is a crucial factor as it regulates the transcription of most of the housekeeping genes and responsible for the most of the DNA regulatory functions. Sigma70 promoter comprises two well-defined short sequences located at-10 and-35 base pairs upstream of TSS, known as pribnow box and-35 region, respectively (Paget and Helmann, 2003). It is essential to identify the promoter regions in a genome, as it can aid in illuminating the genome’s regulatory mechanism and disease-causing variants within cis-regulatory elements. The area of the promoters is of great interest as researchers pay great attention to their importance not only in developmental gene expression but also in environmental response. To control the expression of every gene and transcription unit in the genome, promoters must be precisely identified, and in terms of consensus sequences, promoter sequences may differ and be comparable within and between the different classes of promoters. However, since each promoter often deviates from the consensus at one or more locations, it is still difficult to predict promoters with reliable accuracy (Mrozek et al., 2014, 2016). Moreover, due to the advancement in sequencing technology, the data is growing exponentially, which made the accurate identification of promoter regions in the DNA sequences a difficult task. Of note, the accurate and fast classification of the promoter region is a crucial problem, as the standard experimental procedures are expensive in terms of time, and performance (Bernardo et al., 2009; Lu et al., 2015).

In the past, ample of methods have been developed for predicting sigma70 promoters which are based on different machine-and deep-learning approaches developed using various types of features (Lin and Li, 2011; Song, 2012; He et al., 2018; Liu et al., 2018; Lai et al., 2019; Lin et al., 2019; Liu and Li, 2019; Zhang et al., 2019). IMPD (Lin and Li, 2011), is based on the increment of diversity, which achieved an accuracy of 87.9%. This method was trained on RegulonDB (Gama-Castro et al., 2016) dataset that contains 741 *E. coli* sigma70 promoters. Z-curve-based approach (Song, 2012) attains the maximum accuracy of 96.1% by using a smaller dataset that comprises 576 sigma70 promoters and 1,661 non-promoters. Liu et al. (2018) proposed a two-layer prediction method, named as iPromoter-2L, for the identification and classification of multiple sigma promoters using the multi-window-based pseudo K-tuple nucleotide composition approach and achieved the highest accuracy of 81.68% for sigma70 promoter prediction. 70Propred (He et al., 2018) has incorporated features like position-specific trinucleotide propensity based on single-stranded characteristic (PSTNPss) and electron-ion potential values for trinucleotides (PseEIIP) using benchmark dataset of 741 sigma70 promoters and 1,400 non-promoters from RegulonDB9.0, and reported 95.56% accuracy. iPro70-PseZNC (Lin et al., 2019) is based on a multi-window Z-curve approach and gained the maximum accuracy of 84.5% using the dataset from RegulonDB9.0 (Gama-Castro et al., 2016). iPromoter-2L2.0 (Liu and Li, 2019) is an update of iPromoter-2L, which implemented the combination of smoothing cutting window algorithm and sequence-based features to improve the performance with accuracy 85.94%.

The aforementioned methods are developed using traditional machine learning approaches such as logistic regression (Rahman et al., 2019a), support vector machine (He et al., 2018; Lai et al., 2019; Lin et al., 2019; Liu and Li, 2019; Zhang et al., 2019), random forest (Liu et al., 2018), ensemble of different classifiers (Rahman et al., 2019b). On the other hand, due to the advancement in the computational and sequencing technology, deep convolutional neural network (CNN) based methods have been implemented to develop the prediction methods with the ability to identify the sigma promoters and then determines the different types of sigma promoter sequences such as sigma24, sigma28, sigma32, sigma38, sigma54, and sigma70. Amin et al. proposed a method, iPromoter-BnCNN (Amin et al., 2020), is a branched-CNN based method which utilized the sequence and structural based properties to identify and classify the sigma promoters. Shujaat et al. (2020) introduced pcPromoter-CNN which convert the nucleotide sequence information into one-hot encoding vectors and feed them to convolutional neural network (CNN)-based classifier to predict and determine the sigma promoter classes. Recently, a new method based on the light CNN named as PromoterLCNN was proposed by Hernandez et al. (2022) which also used one-hot encoding representation of nucleotide sequences to predict the sigma promoters using the sequencing information. The correct prediction of sigma70 promoters in the DNA sequences is still a difficult challenge due to the intraclass variation in terms of consensus sequence as sigma70 factor is responsible for the transcription of the most of the regulatory genes. Albeit, number of computational methods are available to predict the sigma70 promoters using the sequence information, but there is a still enough room for the improvement in term of various performance measures.

In the present study, we have developed a computational method called as Sigma70Pred, to classify the sequences in sigma70 promoter and non-promoter. In this study, we have trained and evaluated the prediction model on the benchmark dataset which have been used in ample of previously published methods such as 70Propred, iPro70-FMWin, iPro70-PseZnc, IPMD, iProEP, and iPromoter-FSEn. In order to investigate the validity of the generated model, we have also created the independent dataset with no common sequences with the benchmark dataset. We calculated the performance of the proposed method on the independent dataset and also compared it with the working existing methods. A user-friendly and freely accessible web server and Python and Perl-based standalone software have been developed to serve the scientific community for predicting the sigma70 promoters. Moreover, the same package has also been distributed *via* docker-based technology through GPSRdocker (Agrawal et al., 2019).

## Materials and methods

### Dataset generation

The choice of a standard benchmark dataset is a crucial first step in developing a prediction method. In this study, we have used the high-quality pre-constructed benchmark dataset, which has been used previously published studies such as, 70Propred (He et al., 2018), iPro70-FMWin (Rahman et al., 2019a), iPro70-PseZNC (Lin et al., 2019), iProEP (Lai et al., 2019), IPMD (Lin and Li, 2011), and iPromoter-FSEn (Rahman et al., 2019b). We have trained and tested our models using cross-validation, on the benchmark dataset downloaded from RegulonDB9.0 (Gama-Castro et al., 2016), which is one of the best available databases on bacterial gene regulation in the model organism *E. coli.* K-12. It contains 741 sigma70 promoters and 1,400 non-promoters from the *E. coli.* K-12 genome, and each sequence is of length 81 bp. Due to the lack of sufficient experimentally verified negative data (that is, the locations that are identified not to be transcription start site), randomly generated sequences from the same chromosome have been obtained in the benchmark dataset to generate the non-promoter sequences. As shown by Gordon et al., 81% of the transcription start sites are located at the intergenic non-coding regions and 19% are available in the coding region (Gordon et al., 2003). Therefore, number of methods used the middle regions of long coding sequences of *E. coli.* K-12 genome to create the negative/non-promoter dataset (Shujaat et al., 2020; Hernandez et al., 2022), whereas, other methods used both the coding and non-coding regions to extract non-promoter sequences (Lin and Li, 2011; He et al., 2018; Lai et al., 2019; Liu and Li, 2019; Rahman et al., 2019a,b; Amin et al., 2020). In the benchmark dataset used in this study, half of the negative samples or non-promoter sequences were extracted from the coding and rest half were obtained from convergent intergenic spacers (non-coding regions). In order to validate our model on external or independent dataset, we have extracted the data from RegulonDB 10.8, which comprises 1,134 sigma70 and 638 non-promoters. There is no identical sequence in training and independent dataset. The datasets can be downloaded from our server.

### Overall workflow

**Figure 1:**
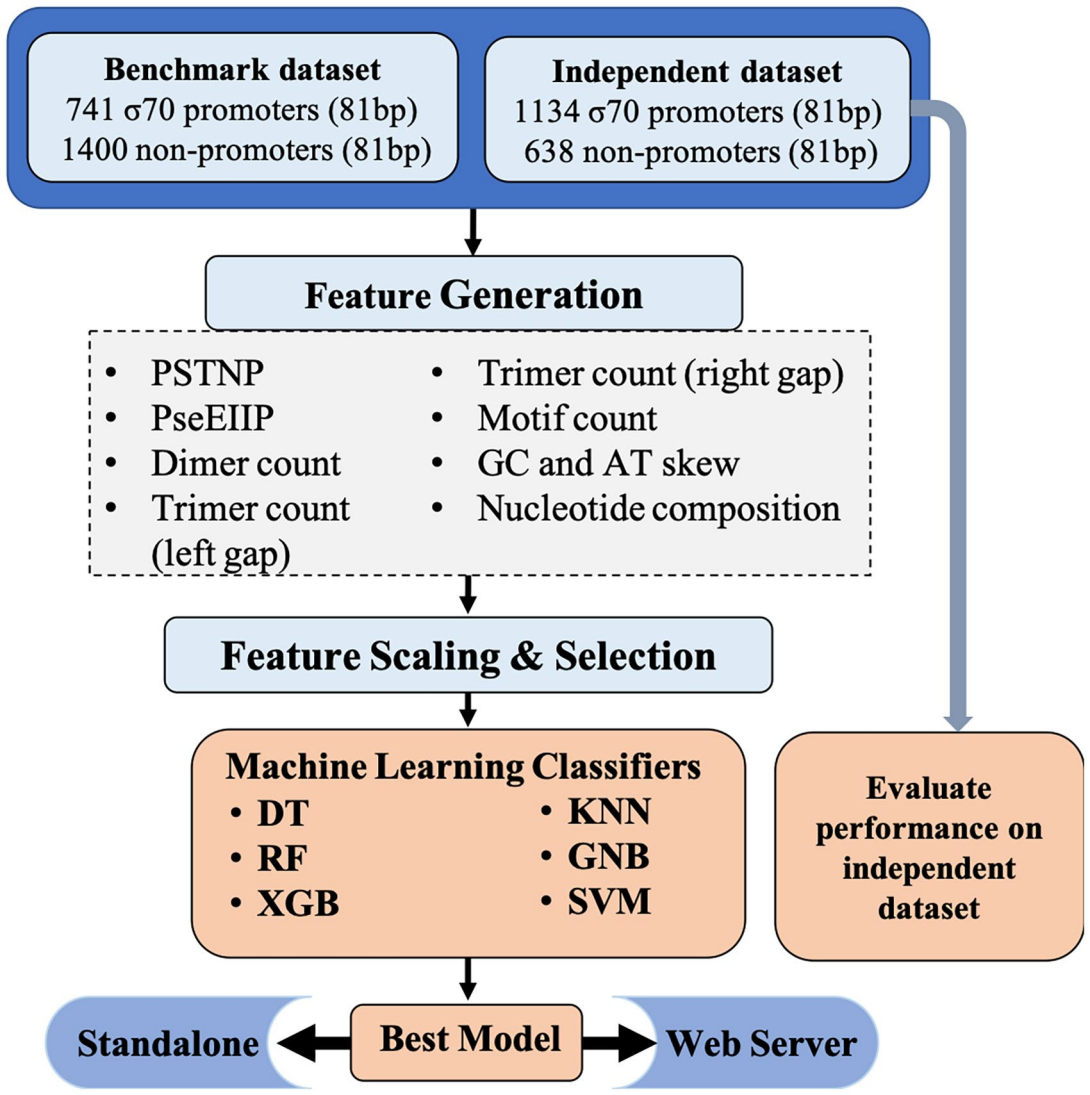
Architecture of sigma70Pred.

The comprehensive workflow for Sigma70Pred is shown in Figure 1.

### Feature generation

We have generated a wide range of features like Position-Specific Tri-Nucleotide Propensity (PSTNPP), Electron-Ion Interaction Pseudopotentials of trinucleotide (EIIIP; He et al., 2018), dimer count, trimer count, motif counts, GC and AT skew (Rahman et al., 2019a), Dinucleotide Auto-Correlation (DAC), Dinucleotide Cross-Correlation (DCC), Dinucleotide Auto Cross-Correlation (DACC; Friedel et al., 2009), Moran Auto-Correlation (MAC), Normalized Moreau-Broto Auto-Correlation (NMBAC; Chen et al., 2015), and Parallel Correlation Pseudo Tri-Nucleotide Composition (PC_PTNC; Liu et al., 2014), which resulted in 8465 features. The aforementioned features were calculated using Nfeature webserver (Mathur et al., 2021) available at https://webs.iiitd.edu.in/raghava/nfeature/. Then, we have used the Min-Max scaler from the scikit-learn library (Pedregosa et al., 2011) to scale down the values of the features, we have constructed. Further, we have implemented Recursive Feature Elimination (RFE; Pedregosa et al., 2011) for the feature selection with logistic regression as the estimator and step-size 10. RFE is a wrapper-style technique, i.e., we have used logistic regression algorithm which is wrapped by RFE, to choose features by iteratively considering smaller sets of features progressively. First, the classifier is trained on the initial set of features and importance of each feature is calculated. Further, the features with least importance are eliminated from the current set of features. This process is recursively repeated on the current feature-set until we are left with the desired number of features. Less number of features can make the models developed using machine learning classifiers, more efficient and effective in terms of space and complexity. It also aid the model to achieve the better predictive performance by avoid learning on the irrelevant input features. Details of each feature and processing of the features are explained in the Supplementary File. The comprehensive details of the top-200 features are reported in Supplementary Table S1, where we have provided the description of each feature along with their mean in sigma70-promoter and non-promoter sequences and value of *p* to check if the difference is significant or not. The features are sorted as per their importance which is calculated using the random forest based classifiers and top-20 features are plotted as per their rank in Supplementary Figure S1.

### Model development

In this study, we developed models for predicting sigma70 promoters using wide range of machine learning techniques such as decision tree (DT), random forest (RF), k-nearest neighbor (KNN), extreme gradient boosting (XGB), gaussian Naïve Bayes (GNB), and support vector machine (SVM; Pedregosa et al., 2011). We got the best performance using SVM based model. Our best model on training dataset was evaluated on independent dataset (obtained from RegulonDB 10.8).

### Cross-validation

In order to avoid the biasness and test the prediction models’ performance, we have implemented five-fold cross-validation. In this approach, the complete dataset is divided into five parts, the model is trained on four out of five parts, whereas the model is tested on the left part, and the performance is recorded. The same process is iterated five times so that each part gets the chance to be used for the purpose of testing. The overall performance is calculated by taking the mean of all five iterations (Patiyal et al., 2020).

### Measures of performance

To assess the performance of generated prediction models, we have used various threshold-dependent and independent parameters. We have considered sensitivity that is, percent of sigma70 samples classified correctly; specificity that is, percent of non-promoter samples classified as negative; accuracy that is, percentage of samples which are correctly predicted by the model; and Matthews correlation coefficient (MCC) that explains the relationship between the observed and predicted value, under threshold-dependent parameters, whereas, in threshold-independent measures, we have considered Area Under the Receiver Operating Characteristics (AUROC) which is the relation between true positive rate and false positive rate. The AUROC was computed using the pROC package (Sachs, 2017) of R. The equations depicting the threshold-dependent parameters are as follows:


(1) $$\mathrm{Sensitivity}=\frac{P_{T}}{P_{T}+N_{F}}\;$$

(2) $$\mathrm{Specificity}=\frac{N_{T}}{N_{T}+P_{F}}\;$$

(3) $$\mathrm{Accuracy}=\frac{P_{T}+N_{T}}{P_{T}+P_{F}+N_{T}+N_{F}}\;$$

(4) $$\mathrm{MCC}=\frac{\left(P_{T}*N_{T}\right)-\left(P_{F}*N_{F}\right)}{\left(P_{T}+P_{F}\right)\left(P_{T}+N_{F}\right)\left(N_{T}+P_{F}\right)\left(N_{T}+N_{F}\right)}\;$$

where, P_T_ refers to number of true positives; P_F_ refers to number of false positives; N_T_ refers to number of true negatives; and N_F_ refers to number of false negatives.

## Results and discussion

### Compositional analysis

**Figure 2:**
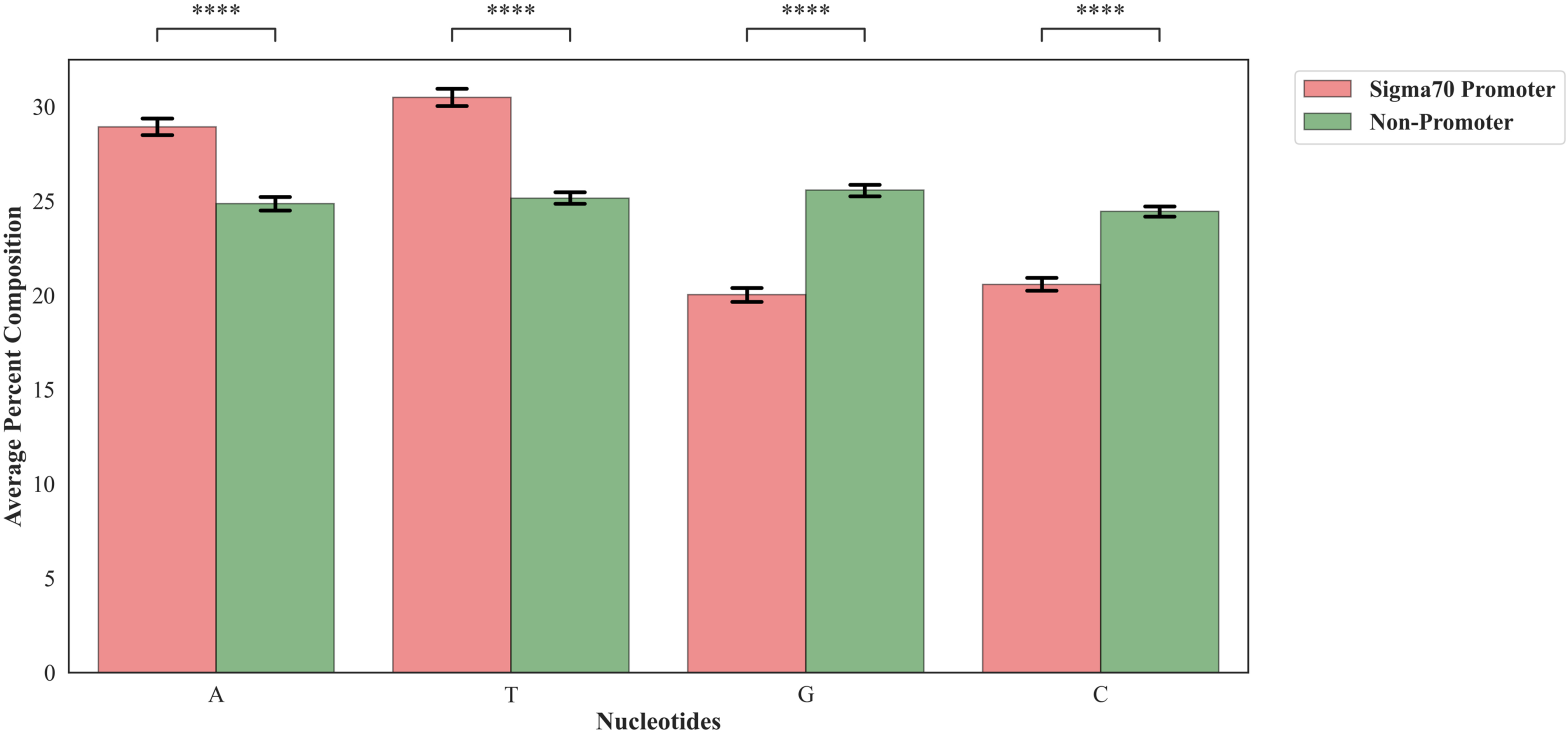
Mono-nucleotide composition of sigma70 promoters and non-promoters. ****p < 0.0001.

In order to assess the proportion of the nucleotides in the sigma70 promoter and non-promoter, we have calculated the mono-nucleotide composition. As shown in Figure 2, nucleic acid adenine and thymine are abundant in sigma70 promoter sequences, whereas cytosine and guanine are higher in percentage in the case of non-promoter sequences.

### Position conservation analysis

**Figure 3:**
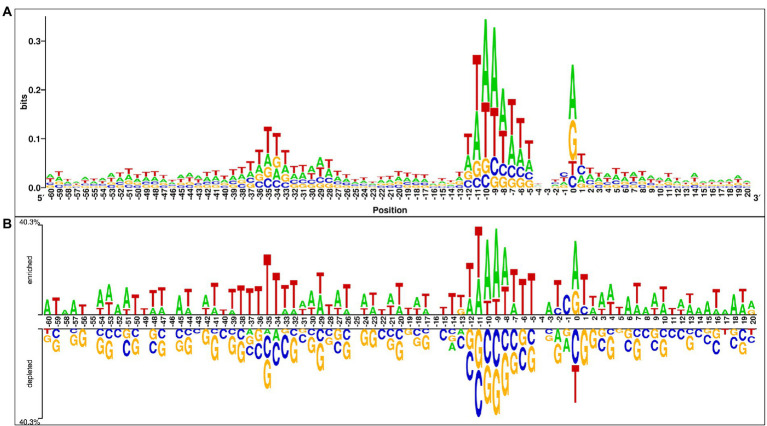
Positional preference analysis **(A)** One sample logo exhibiting nucleotide preference in sigma70 promoter sequences at different positions. **(B)** Two sample logo to exhibit the preference of nucleotides at each position in sigma70 promoter sequences with respect to non-promoter sequences.

In this analysis, we explored the preference of each nucleotide at each position of the sigma70 promoter sequences. For the same, we have created the one-sample and two-sample logo using WebLogo (Crooks et al., 2004) and Two Sample Logo (TSL) tool (Vacic et al., 2006). One Sample logo reports the abundance of nucleotides at each position in a single dataset (i.e., positive/negative dataset), whereas TSL takes two files as input (i.e., positive dataset and negative dataset) to exhibits the preference of nucleotides in the positive dataset in comparison to the negative dataset. Therefore, we have provided sigma70 promoter sequences in the FASTA format to WebLogo tool to generate the one-sample logo, and provided both the files, i.e., sigma70 promoter and non-promoter sequences in the FASTA format to TSL tool. Figure 3A represents the one sample sequence logo and Figure 3B exhibits the two-sample logo for sigma70 promoter sequences. In Figure 3A, consensus short sequences “TATAAT” and “TTGACA” at position-10 and-35, respectively, is blurred due to the variability in the spacing between these regions (Shultzaberger et al., 2007), as we have taken all the sequences to generate the sequence logo. However, the region around-10 and-35 is abundant with the nucleotides involve in the consensus sequences at-10 and-35. As shown in Figure 3B, sigma70 promoter sequences are enriched in “A” and “T” nucleotides at most of the positions, whereas, depleted in nucleotides “G” and “C.” “T” is most abundant nucleotide at positions −59, −56, −50, −49, −40, −38 to-34, −28, −22, −19, −15, −14, −6, −5, +5, and + 11. Whereas nucleotide “A” is preferable at positions −60, −58, −57, −52, −45, −3, +6, +8, +14, +15, +17, and + 18 in the sigma70 promoter sequences. On the other hand, at positions −13, 0, and + 20 nucleotide “G” is also preferred, and positions −2, −1, and + 1 are also occupied with nucleotide “C.” Whereas, on the rest of the positions, both “A” and “T” are the most abundant nucleotides in the sigma70 promoter sequences, as shown in Figure 3B. In order to represent the-10 and-35 consensus sequence, we have generated the motif using MEME software (Bailey et al., 2009) and highlighted the sigma70 promoters’ conserved sequences “TATAAT” and “TTGACA” in Supplementary Figure S2.

### Performance of machine learning classifiers on benchmark dataset

Initially, we have generated more than 8,000 nucleotide-based features, and then selected 200 most relevant features after applying feature scaling method min-max scaler and feature selection method RFE. Using these selected features, we have generated various models by implementing various machine learning techniques. To compare the performance of each generated model, we have calculated different performance measures as reported in Table 1. The model developed using SVM-based classifier performed best among all the other classifiers with 97.38% accuracy, 0.996 AUROC, and 0.94 MCC on the benchmark dataset.

**Table 1:** Performance of various machine learning classifiers on benchmark dataset.

Classifier	Sensitivity	Specificity	Accuracy	AUROC	MCC
DT	74.49	87.14	82.77	0.808	0.62
RF	92.04	91.57	91.73	0.977	0.82
XGB	91.90	92.14	92.06	0.980	0.83
KNN	90.15	91.79	91.22	0.958	0.81
GNB	88.66	88.71	88.70	0.955	0.76
**SVM**	**97.44**	**97.36**	**97.38**	**0.996**	**0.94**

The values in the tables are in bold to represent the best performing classifier or method.

### Performance comparison with existing methods on benchmark dataset

There are ample of methods which are trained and evaluated on the same benchmark dataset such as, 70ProPred (He et al., 2018), iPro70-FMWin (Rahman et al., 2019a), iPro70-PseZNC (Lin et al., 2019), Z-Curve (Song, 2012), IPMD (Lin and Li, 2011), iProEP (Lai et al., 2019), and iPromoter-FSEn (Rahman et al., 2019b). Out of all the considered methods, four methods such as 70Propred, iPro70-PseZnc, Z-curve, and IPMD were not available or working. Therefore, for such methods we have considered the performance reported by the authors in their respective articles for comparison. For rest of the methods, we have predicted the class by providing the benchmark dataset as input and calculated the performance measures based on the predictions made by the respective methods. We have compared the performance of Sigma70Pred with sigma70 promoter prediction methods and found out that Sigma70Pred has outperformed all the considered methods, as shown in Table 2. In terms of AUROC, out of the all the methods developed on the same benchmark dataset, 70Properd attained the second highest performance with AUROC of 0.990, followed by iPro70-FMWin with AUROC of 0.960.

**Table 2:** Comparison of performances of our model with existing method on benchmark dataset evaluated using cross-validation technique.

Methods	Sensitivity	Specificity	Accuracy	AUROC	MCC
Sigma70Pred	**97.44**	**97.36**	**97.38**	**0.996**	**0.943**
iPro70-FMWin	83.81	95.07	91.17	0.960	0.803
70ProPred^*^	92.40	96.90	95.30	0.990	0.897
iPro70-PseZNC^*^	80.30	86.80	84.50	0.909	0.663
Z-Curve^*^	74.60	79.50	77.80	0.848	0.527
IPMD^*^	82.40	90.70	87.90	–	0.731
iProEP	89.52	64.03	76.88	0.654	0.554

* Reported by the authors in the manuscript. The values in the tables are in bold to represent the best performing classifier or method.

### Performance comparison on independent dataset

In order to evaluate the proposed method’s robustness and performance, we have also investigate the performance of our proposed model on the independent dataset of DNA sequences extracted from RegulonDB 10.8. We have also considered the existing methods for performance comparison on the independent dataset, which were trained and evaluated on different datasets such as MULTiPly (Zhang et al., 2019), iPromoter-2L (Liu et al., 2018), and, iPromoter-2L2.0 (Liu and Li, 2019). Moreover, to compare the efficiency of our generated model with deep-learning based classifiers, we have compared the performance with methods like iPromoter-BnCNN (Amin et al., 2020), pcPromoter-CNN (Shujaat et al., 2020), and PromoterLCNN (Hernandez et al., 2022). We have calculated the different performance measures for all the working sigma promoter predictors. The results on the independent dataset showed that our proposed model is quite robust towards the unseen data and performs well on it (Table 3). It also implies that our SVM model is significantly free from bias and overfitting on training dataset. As shown in Table 3, method named “MULTiPly” considered for the comparison which is not able to produce the results, therefore we have reported the performance achieved by the authors in this method. For comparison, we have considered the methods developed using machine-learning as well as deep-learning based classifiers. As exhibited in Table 3, SVM-based model developed on top-200 features in Sigma70Pred outperformed all the existing approaches in terms of each performance measure. Two-layer predictor method iPromoter2L-2.0 achieved the second highest accuracy of 83.36% on the independent dataset, followed by light-CNN based method PromoterLCNN with 79.56% accuracy.

**Table 3:** The performance of existing methods on independent dataset.

Methods	Sensitivity	Specificity	Accuracy	AUROC	MCC
Sigma70Pred	**91.45**	**88.56**	**90.41**	**0.953**	**0.794**
iPro70-FMWin	84.12	86.67	85.04	0.921	0.693
iProEP	84.50	53.83	69.30	0.541	0.404
MULTiPly^*^	90.43	76.93	84.91	–	0.685
iPromoter-2L	86.21	72.81	79.56	–	0.601
iPromoter-2L2.0	88.72	77.91	83.36	–	0.674
iPromoter-FSEn	68.76	68.16	68.46	0.751	0.369
iPromoter-BnCNN	80.64	72.70	76.71	–	0.543
pcPromoter-CNN	81.44	61.07	71.35	–	0.445
Promoter-LCNN	88.77	70.15	79.54	–	0.604

* Reported by the authors in the manuscript. The values in the tables are in bold to represent the best performing classifier or method.

### Implementation of model in web server

In order to serve the scientific community, we have also developed the webserver Sigma70Pred by implementing our best model to predict the sigma70 promoters. The web server consists of three modules namely “Predict,” “Scan,” and “Design.” Our final model is based on SVC, it calculates SVC score for a sequence. SVC score is proportional to probability of correct prediction to promoter. SVC score varies from 0 to 1, higher the SVC score chances are higher that sequence is a sigma70 promoter. To provide balance between sensitivity and specificity, we provide default threshold. User may select desire threshold depending on their need. The detailed description of each module is as follows:

#### Predict

This module allows users to classify the submitted sequence as sigma70 promoter or non-promoter. There is a restriction of length in this module, as the model is trained on sequences with length 81 bp, hence if the submitted sequence is having a length less than 81, “A” will be added as the dummy variable and then, the sequence will be classified into one of the class, and if the length is greater than 81, only first 81 nucleotides will be considered for prediction. The user can submit sequences in either FASTA or single line format, and can select the desired threshold as SVC score above which the sequence will be classified as sigma70 promoter, otherwise non-promoter. The user can either provide single or multiple sequences, and can also upload the text file containing sequences. The output page displays the results in the tabular form, which is downloadable in the csv format.

#### Scan

Scan module allow users to scan or identify the sigma70 promoter region in given genome. This module does not have any length restriction as in the “predict” module. In this module, overlapping patterns of length 81 will be generated from submitted sequences and then used for prediction. The user can provide single or multiple sequences either in FASTA or in single line format. The user is also allowed to upload the sequence file. The output result will exhibit the overlapping patterns of length 81 with the prediction as promoter or non-promoter. The result is downloadable in the csv format.

#### Design

Design module allow users to identify the minimum mutations that can convert the sigma70 promoter into non-promoter or *vice-versa*. This module also has the restriction of sequence length 81, as it generates all the possible mutants by changing nucleotides at each position and then make the predictions based on the selected threshold. Since, generating all possible mutants is a time and computational expensive process, hence only one sequence is allowed at a time. The output page displays all the possible mutants with its prediction as promoter or non-promoter in tabular form which is downloadable in csv format.

#### Standalone

We have also developed Python and Perl-based standalone package, which is downloadable from URL: https://webs.iiitd.edu.in/raghava/sigma70pred/stand.html. The advantage of this module is that, it is not dependent on the availability of the internet, the user can download these standalone on their local machines and can use all the aforementioned modules. This module also take the input as single or multiple sequences in a file, in either FASTA or single line format. The output will be stored in the user-defined file in the comma separated value format.

## Discussion

The expression of genes decides the cell’s fate, which is regulated by the promoter regions present upstream of the transcription start site (Atkinson and Halfon, 2014). The interaction between the promoter region and the holoenzyme, switch on or off the expression of the respective genes. Various sigma factors are associated with the holoenzyme responsible for different functions, such as regulating nitrogen levels, controlling stationary phase genes, etc. (Paget, 2015). One of the essential sigma factors is sigma70, as it regulates the expression of most of the housekeeping genes required for the cell’s survival (Paget and Helmann, 2003). The accurate identification of the promoter regions associated with the respective sigma factors may help in the understanding of the regulatory mechanism, which can further be exploited to treat diseases caused by the disease-causing variants. The recognition of the promoter regions has been an important aspect of gene structure recognition and it is also the fundamental problem in building a network of gene transcriptional regulation. However, the experimental methods to identify the promoters are laborious, expensive, and time-consuming. On the other hand, computational approaches are reliable and fast with equivalent accuracy. Although, several methods have been developed in the past for the prediction of sigma promoters in the DNA sequences based on machine-learning (Lin and Li, 2011; Song, 2012; He et al., 2018; Liu et al., 2018; Lai et al., 2019; Liu and Li, 2019; Zhang et al., 2019) and deep-learning approaches (Amin et al., 2020; Shujaat et al., 2020; Hernandez et al., 2022), but the accurate identification of the sigma promoters remained a strenuous task due to the inter-and intra-class similarities and variations in the different sigma-specific promoter sequences (Zhang et al., 2019). It has been seen in the past that promoter sequences often differ at one or more locations from the consensus sequences (Mrozek et al., 2014, 2016), which makes the task of prediction of sigma70 promoters more difficult as sigma70 factor specific promoters are responsible for the transcription of most of the genes in prokaryotic genome. Moreover, the exponential increase in the data of promoter sequences due to the advancement in the high-throughput sequencing technology, also increased the level of difficulty in the identification of sigma70 promoter regions in the DNA sequences. Therefore, an accurate and robust method is required that can distinguish the sigma70 promoter sequences from the non-promoter sequences.

To understand the preference of nucleotides in the sigma70 promoter sequences, we have conducted the compositional and positional preference analysis for the sigma70 promoter sequences (Figures 2, 3). The compositional analysis showed that nucleotides “A” and “T” are in higher abundance in sigma70 promoter sequences in comparison with non-promoter sequences. For positional preference analysis, we have generated one-sample and two-sample logo using WebLogo and TSL logo tool. In one-sample logo, the preference of nucleotide at each position is shown in Figure 3A, however, the consensus sequences at position-10 and-35 is not clear. As shown by Shultzaberger et al. (2007) the gap between the regions-10 and-35 is not fixed, it varies from promoter to promoter. Therefore, they have shown the consensus sequences in their Figure 2 of the article at-10 and-35 regions in the form of sequence logos by vary the spacing between 21 and 26. On the other hand, we have generated the sequence logo by taking all the sigma70 promoter sequences without considering the variability in the spacing between the-10 and-35 regions. Whereas, in Figure 3B, we have represented the two-sample logo, by considering the sigma70 promoter and non-promoter sequences. It corresponds with the compositional analysis that most of the positions in the sigma70 promoter sequences are abundant in nucleotides “A” and “T” in comparison to the non-promoter sequences.

There are different methods which are specific to the classification of sigma70 promoters (Lin and Li, 2011; Song, 2012; He et al., 2018; Lai et al., 2019; Rahman et al., 2019a,b) whereas others are developed for the identification and classification of different sigma promoters such as sigma24, sigma28, sigma32, sigma38, sigma54, and sigma70 (Liu et al., 2018; Liu and Li, 2019; Zhang et al., 2019; Amin et al., 2020; Shujaat et al., 2020; Hernandez et al., 2022). In this study, we have also developed a bioinformatic-ware to classify the sigma70 promoters using only sequence information. The models were trained and evaluated using the nucleotide sequences of length 81 bp in the benchmark dataset retrieved from RegulonDB9.0 (Gama-Castro et al., 2016), which consists of 741 sigma70 promoters and 1,400 non-promoters. Initially, we calculated more than 8,000 features for each sequence, which were further processed using min-max scaling and top-200 most relevant features were selected using RFE feature selection technique. Further investigation was performed on these selected features. Then, we have implemented six different machine-learning classifiers to develop the prediction models on the selected features. The SVM-based model outperformed all the other classifiers with AUROC of 0.996 on the benchmark dataset (See Table 1). To understand the advantages and disadvantages of a new method, it is important to compare the proposed method with the already existing methods. We have considered already existing methods, some of them were non-functional, hence we have considered the performance reported in their respective articles for those methods. For rest of the methods, we have used the benchmark dataset to evaluate and compare the performance. Our proposed method has outperformed the methods developed on the same benchmark dataset, as shown in Table 2. Further, in order to check the efficiency of the proposed method, the generated model was evaluated and compared with existing methods using the unseen independent dataset, where sigma70pred outperformed the existing working method with AUROC of 0.953 (see Table 3). This comparison signified that our feature-set of 200 features is more effective to identify the sigma70 promoter sequences.

To understand the reason behind the wrong predictions made by our proposed model, we have selected all the sigma70 promoter sequences which were predicted as non-promoter, and provided them to the other existing sigma promoters predicting approaches. We found that most of the selected sequences were also wrongly predicted by other methods. Further, we checked the similarities of these sequences with the benchmark dataset using the “blastn” approach. For that, we have created a customized database using the sequences in the benchmark dataset by implementing the “makeblastdb” module of the BLAST program version 2.1.2. Then, we hit the wrongly predicted sequences to the customized dataset and considered the top-hit for further analysis. We have observed that most of the top-hit were non-promoter sequences, i.e., sigma70 promoter sequences in the independent dataset share similarity with the non-promoter sequences in the benchmark dataset. The negative data in the benchmark dataset used by several studies, was generated randomly from the coding and non-coding regions of *E. coli*. K-12 genome. Therefore, there is a need to develop the experimentally verified non-promoter sequence dataset to improve the overall performance and efficiency of the prediction methods.

Moreover, Shimada et al. (2014) introduced the whole set of constitute promoters which was defined as the promoters recognized *in vitro* by the RNA polymerase RpoD holoenzyme without needing the additional supporting proteins. They have provided the list of the promoter sequences along with the genes which is controlled by the respective promoters. In order to investigate the efficiency of the our proposed method to classify the constitutive promoters, we have extracted the sequences from RegulonDB (Tierrafria et al., 2022) and colibir (Medigue et al., 1993) and used them for the prediction. We were able to extract the 329 promoter sequences, which were then submitted to the “predict” module Sigma70Pred web server with default parameters. 268 (81.46%) out of 329 were predicted as sigma70 promoters at the default threshold, which was increase to 276 (83.89%) on dropping the threshold to 0.2. The result on each promoter sequence is reported in Supplementary Table S2 along with the SVC score. These results signify that our proposed model is able to classify the constitutive promoters with reliable accuracy.

Sigma70Pred offers a web server and standalone packages to predict the sigma70 promoters using sequence information. This method uses 200 different optimal features, and we assume that our features have more capability to classify sigma70 promoters. Sigma70Pred provides three major modules: predict, scan, and design. As the application of our method, the user can scan the entire prokaryote genome to identify the sigma70 promoter using the scan module. By using the design module, the user can also determine the minimum number of mutations required to exploit the sigma70 promoter regions, i.e., either induce or deteriorate the capability of the sigma70 promoter. As compared to the existing methods of predicting sigma70 promoters, Sigma70Pred produced commending outcomes. We believe that Sigma70Pred will play an essential role in the area of genomic analysis.

## Data availability statement

Publicly available datasets were analyzed in this study. This data can be found at: https://webs.iiitd.edu.in/raghava/sigma70pred/data.html.

## Author contributions

GR conceived the idea and supervised the entire project. NS, MA, and DP collected and curated the datasets. SP, NS, MA, and DP wrote all the in-house scripts, performed the formal analysis, and developed the prediction models. SP developed the web interface and standalone. SP and GR prepared all the drafts of manuscript. All authors contributed to the article and approved the submitted version.

## Conflict of interest

The authors declare that the research was conducted in the absence of any commercial or financial relationships that could be construed as a potential conflict of interest.

## Publisher’s note

All claims expressed in this article are solely those of the authors and do not necessarily represent those of their affiliated organizations, or those of the publisher, the editors and the reviewers. Any product that may be evaluated in this article, or claim that may be made by its manufacturer, is not guaranteed or endorsed by the publisher.
